# Effect of Organic Compounds on the Special Properties and the Microstructure of Autoclaved Brick

**DOI:** 10.3390/ijerph20043490

**Published:** 2023-02-16

**Authors:** Ryszard Dachowski, Anna Stepien

**Affiliations:** Civil Engineering and Architecture Department, Kielce University of Technology, 25-314 Kielce, Poland

**Keywords:** humus, silicates, bricks, ecology, organic remains

## Abstract

After a long decomposition process, organic matter turns into humic substances. In humus, carbon dioxide (CO_2_) bound in photosynthesis is brought back to the soil, where it should be used by its ecosystem. This is important because similar relationships are found in modern concretes and concretes designed with the use of geochemical modeling (possibility of the C-S-H phase for storing harmful substances). The aim of the article was to investigate the possibility of using humus (Humus Active-HA) and vermicompost (Biohumus Extra Universal-BEU), i.e., organic matter resulting from a long process of biological decomposition in the production of autoclaved bricks containing only ecological materials, i.e., sand, lime, and water. Tests of compressive strength, density, microstructure based on SEM, XRD, and micro-CT analysis were performed. The results of the research indicate that humus and vermicompost can be successfully used in their production. The paper compares traditional products and products made of raw material mass containing 3%, 7%, and 11% of humus and vermicompost, using the apparatus of mathematical experiment planning. Compressive strength, volumetric density, water absorption, and wicking, porosity, and material microstructure were tested. The best results were obtained for samples with the addition of 7% humus and 3% vermicompost. The compressive strength increased to 42.04 MPa (compared to standard bricks, whose strength is 15–20 MPa), and the bulk density increased by about 55%, to the value of 2.11 kg/dm^3^, which indicates the densification of the material’s microstructure. They were characterized by the highest compressive strength, moderate water absorption, and a high proportion of closed pores in the sample.

## 1. Introduction

The construction industry focuses on exceptional large social benefits, such as raising living standards, economic and environmental, as well as having a noticeable impact on the ecosystem and nature. As part of sustainable development, construction companies, apart from construction activities, must now also contribute to the improvement of environmental and ecological conditions (e.g., recycling and sustainable activities for natural processes of environmental restoration). Environmental protection, proper waste management, waste processing, and all modifications in the industrial sphere constitute the basis of the present ecology and sustainable construction. Among the modifications, one of the interesting solutions is the use of humus in building materials. Humus is a stable form of added plant and animal residues. After a long decomposition process, organic matter turns into humic substances. Naturally occurring humus occurs in a smaller amount in the soils of arid and semi-arid regions [1]. In order to determine the applicability and then the action of organic compounds on the properties of building materials, attention was focused on silicate products. Silicates are the most environmentally friendly products. They are produced exclusively from sand, lime, and water. These materials are the most ‘clean’ in terms of ecology. Every year, the Central Laboratory of Radiological Protection in Poland sends updated data on concentrations of the radionuclides potassium-40, radium-226, thorium-228, and activity indicators f1 and f2 for selected raw materials of natural origin, industrial origin, and building materials to the Central Statistical Office. These results show that silicate products have the lowest concentrations of radioactive elements [2].

The global market for silicate products in the world continues to grow. Global silica brick high-temperature hot air furnace volume for 2030, compared to 2017, will probably increase by half [3]. North America, Europe, and Asia-Pacific have the largest share in this industry. For years, there was a belief that silicate products are a material of the so-called “Wet”, which is related to the quick absorption of water by the silicate. However, a number of standards contain information that silicate products absorb water only in 16% (in relation to their mass), while the water absorption of the remaining part of building materials used for the construction of external walls reaches the value of 24% (in relation to the mass). In line with the policy of sustainable development, i.e., a phenomenon that became common in Poland over the last 8 years (the subject was discussed for the first time at the Concrete Days in 2010 in Wisła, Poland), modifications are made to the currently used building materials, ranging from concrete of various classes, quality, and purpose of the remaining groups of materials used in construction (also products used for making insulation, thermal insulation systems, woodwork, etc.). The disposal of waste products or the possibility of using organic components, as well as the production of materials capable of CO_2_ accumulation or even those conducive to activities aimed at reducing the carbon footprint, became a significant problem. The further analysis is the basis for an unequivocal determination of which sand–lime mass modifier has a beneficial effect on the performance properties of silicate products. This is of particular importance in the foundation zone of a building, where the material is exposed to moisture from the ground.

Another aspect is the material’s ability to absorb CO_2_. In this context, we return to geology and soil observation. The ability of soils to adsorb carbon is reduced by factors such as: leaving the soil without plant cover, using chemicals, destroying the structure with heavy equipment, urbanization, etc., and similar phenomena can be found during the production and operation of concrete. Carbon dioxide (CO_2_) bound in photosynthesis is returned to the soil, where it should be used by its ecosystem. In order for the carbon contained in CO_2_ to be effectively and permanently bound in the soil, a series of complex physico-biochemical processes must take place, carried out mainly by bacteria and higher organisms [4]. In the case of autoclaved materials, this factor may be hydrothermal processes. Therefore, an analysis of the possibility of using waste or organic materials in the lime–sand mixture was carried out. The total of all organic substances present in soil is often expressed as a percentage of carbon, since carbon is the basic building block of organic material [5]. These substrates were analyzed for their content, i.e., they are components rich in elements and chemical compounds (including aluminum and phosphorus), as well as in microorganisms. Humus and vermicompost were subjected to a multi-criteria analysis. Technical analysis of patents and literature in databases showed that all modifications of biohumus or humus, or their use, are mostly related to the agricultural sector, although there are also applications in construction. They concern fertile land, i.e., soil. Components are introduced into humates that improve their properties from the biological point of view, i.e., improve the digestibility of food products by plants. These are aluminum compounds, silicon, and calcium carbonate. With biohumous plants, earthworms process biological mass at the same time. In total, three aspects of application can be distinguished: agriculture, ecology, and construction. In the first group, the most important, we separate a subgroup—improvement of physicochemical properties/quality/soil fertility (there is an addition of biohumus or vermicompost to the soil in order to increase the production of expensive saffron. These microorganisms can also be useful as silicate modifiers [6,7]. Until the autoclaving stage, the addition of a soil bioinoculant may prove useful for the humification of silicate mass. These compounds can be used as a liquid substrate [8] for the evaluation of compost fertilizers for the content of humus compounds; and these data are the basis for the modification of silicate bricks [9,10,11,12,13], to evaluate the effect of potassium humus used by treating soil seeds on cotton productivity and thus the quality characteristics of the fibers thus produced (microorganisms of humus and cotton fibers can also be the basis for using silicate products as modifiers) [1].

The second group is ecology, in which the remediation of land contaminated with diesel oil is distinguished by cultivating plants used for the production of energy or bioproducts, with the humus in this group being enriched with biochar in order to improve the morphological and physiological parameters of the cultivation. Plants enriched with carbon can be used as a substrate for silicate products [14], and thanks to the use of microelements contained in the soil, we contribute to reducing the carbon footprint [15,16]. Another important aspect of this method of using vermicompost is the decrease in the amount of worms as a result of agricultural intensification [17], and at the same time with vermicelli, the biological mass is processed by earthworms, thanks to which, the amount of CaO, which is beneficial in the autoclaving of silicates, is increased. It should be emphasized that in contaminated areas (e.g., Asia) 21 atypical forms of humus were found that do not fit the European classification, the extinction of earthworms caused by soil toxicity, and consequently led to the transformation of humus profiles [18]. Development of an algorithm for the amount of lime added to sandy and humus-poor soil (north-eastern Germany) is based on the determination of the optimal soil pH and the soil response to lime, which is described by the basic neutralizing capacity (BNC), showing different concentrations of Ca(OH)_2_; calcium carbonate is an important chemical compound here, improving the functional properties of silicates [19].

**Figure 1:**
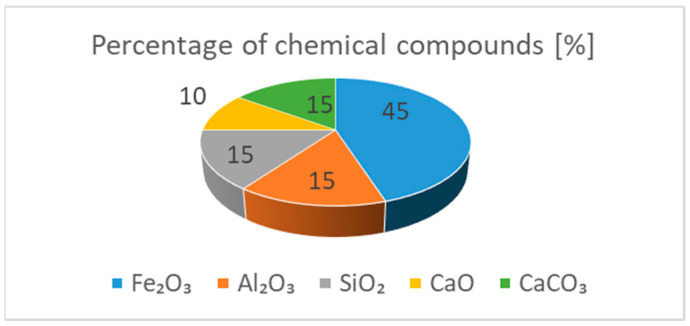
Percentage of chemical compounds in vermicompost [22].

The third group is construction, which at the moment is a very small group. The desirability of using organic compounds in our case can also be supported by analyzing, inter alia, the patents described below. In the patent (Watabe Akinori, Fukuda Kuni, Saito Takao. Patent 62-090530 (1987)) a soil activator is disclosed to increase the amount of humus in the soil during fertilization and to promote the production of porous limestone [20]. In the patent (Charier G.A.; Le Guerinel H.; Raimbault S.; Jeanneau S. Patent FR2823502 (A1)), a substance improving soil structural properties, containing a mixture of lime, household waste, and plant compost was modified [21]. The patent (Nebehaj I., Venesz B., Odor G., Csapo F., Szuecs L. Patent HUT57688 (A)) presents a method of producing vermicompost with an increased content of microelements. Nebehaj I. et al. (patent HUT57688 (A)) increase the level of micronutrients in vermicompost by introducing red sludge (including Fe_2_O_3_—40 ÷ 45%, Al_2_O_3_—10 ÷ 15%, SiO_2_—10–15%, and CaO—6 ÷ 10%) and CaCO_3_ (Figure 1) [22] from organic materials of plant origin. These compounds have a positive effect on the physical and mechanical properties of silicate products. It is also worth mentioning the team of scientists [23,24,25,26,27,28,29] from the construction industry, who introduce a number of different additives to the silicate mass (including basalt, glass sand, barium, and lithium), also improving the physical and mechanical properties of silicate products.

There is a group of scientists [30,31,32,33] that tries to improve the process of sand–lime brick production or conducts research works aimed at improving the physical and mechanical properties of the products in question. Several variants of the proposed changes are presented below, although not all of them guarantee the receipt of the so-called natural product, which is silicate.

Pytel and Małolepszy J. from AGH in Krakow (PL) [30] propose introducing the so-called post-chrome mud. However, this proposal is not entirely accurate, because the chromium mud is a waste material, and in order to use it for the production of silicate products, taking into account their ecology and the natural nature of the substrates, it is necessary to neutralize toxic chromium compounds beforehand, and only in the next stage to introduce such a modifier to sand–lime mass. However, this is a time-consuming and low-cost procedure, although it can be considered due to environmental protection and the use of waste substances. The team of researchers Malhotra S.K., Tehri S.P. [31], in their research, deal with sand–lime mass, but on the basis of slags (lime–slag mass (20–50%) with sand (50–80%)). Such modification of the mass, however, causes a decrease in compressive strength within the range of 8–15 MPa (the strength of traditional silicate reaches 20 MPa). Witthohn M., Wittenborn, D.E., and Klemm R. developed bricks with increased heat capacity based on a lime–silicate mass (silicate or preferably, according to the inventors, cellular concrete). This modification is to consist of introducing a fusible heat-accumulating material into the gaps in the porous product, which consequently remains permanently in the modified material. The presented invention, however, has a serious drawback, because such modification of the building material disturbs or even significantly reduces the water vapor permeability, as the binder layer blocks the water vapor exchange of the material with the environment. This further causes deterioration of the microclimate inside buildings made of products modified in this way. It is similar with the above-mentioned items. The production costs of modified products according to the cited inventions exceed the costs of a standard product [32].

There was an article [33] devoted to TBM-EPB tunneling technology. In tunneling machines (TBM) using the equivalent pressure method, there is a problem of biodegradation of the mixtures used. The effects of three mixtures and two inoculums (soil humus and Bacillus Clausii) were investigated. In the case of Bacillus Clausii, it was not possible to compare the different formulations in a short time. Acceptance of soil humus met only the criteria of a quick test, while Bacillus Clausii, as a specific inoculum, may meet the criteria of reproducibility of the results. Kinetic studies of organic carbon removal were also carried out. It is a contribution to the use of soil humus with specific chemical properties in the production of silicate products.

Summarizing this introduction, we come to the conclusion that the purpose of the article was to investigate the possibility of using humus and biohumus, i.e., organic matter resulting from a long process of biological decomposition in the production of autoclaved bricks containing only ecological materials, i.e., sand, lime, and water.

## 2. Materials and Methods

**Figure 2:**
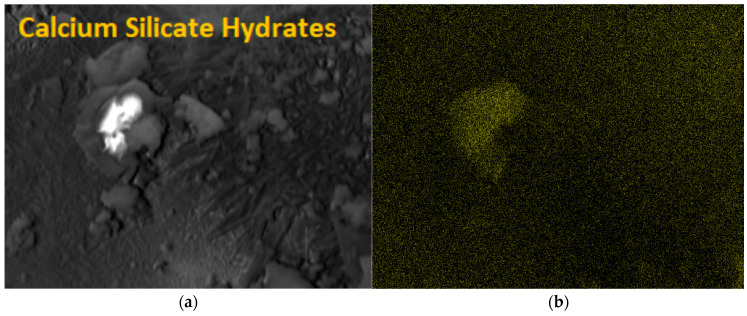
Photo of the microstructure of a silicate product made with a scanning electron microscope (SEM): (**a**) image of a sample of biohumus-modified silicate brick; (b) description of what is contained in the second panel. Figures should be placed in the main text near to the first time they are cited. A caption on a single line should be centered.

Sand–lime products are elements that are formed in a hydrothermal process, which means that they are autoclaved at a temperature of usually 200–203 °C. The introduction of organic compounds, humus, or biohumus at the production stage of the products in question is safe precisely because of the high temperatures. Four types of humus and vermicompost were tested. The tests were carried out on the basis of microscopes: Kozo Optics XJS 800 FL and LSM 700 by ZEISS, on the basis of which the composition of the tested substrates was identified. The research shows the presence of plant debris, and also microorganisms in vermicompost. The composition of a lime–sand mixture modified with a selected organic modifier in a liquid form was developed in order to improve the modification process—the filler can be easily combined with water while mixing the lime–silicate mass (Figure 2a,b). It is one of the key and important conditions for the company in terms of the efficient use of energy and the proper production process of sand–lime brick. An important issue is also the possibility of using organic compounds, humus or biohumus as a filler of sand–lime mass, which would reduce the consumption of sand used in the production of silicate products. A total of 6 ÷ 22% of the mix modifier in liquid form was added to the prepared lime–sand mixture. Due to the liquid form of the applied modifier, it is not advisable to use water for the mixture. The resulting modified mixture is pressed and then autoclaved. The resulting material should be characterized by lower water absorption (up to 16% absorption in relation to the weight of the product, and optimally up to 10%) and increased compressive strength (due to the possibility of its possible use in foundation works). Possible technological and material solutions should take into account the properties of the lime–sand mixture, its optimal composition after consultations and tests, and the final amount and method of the modifier dosed. The following standards were used: CEN. PN-EN 772-1: 2011E, CEN. PN-EN 772-13, CEN. PN-EN 1996-2: 2010, and CEN. PN-EN 771-2 [34,35,36,37].

### 2.1. Research Plan

In order to select a modifier for sand–lime mass, a multi-criteria technical and economic analysis was performed. For an in-depth assessment of the features of additives affecting the optimization result, multi-dimensional analysis methods were used [38,39,40].

**Figure 3:**
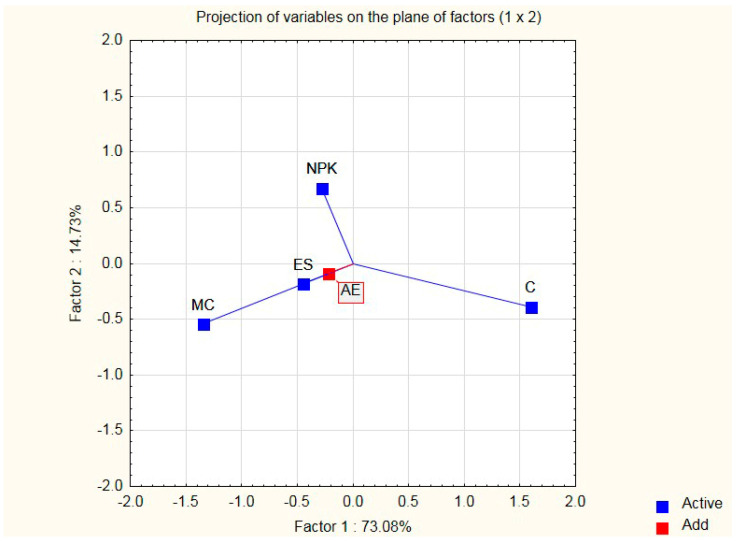
Projection of variables on the plane of variables.

**Figure 4:**
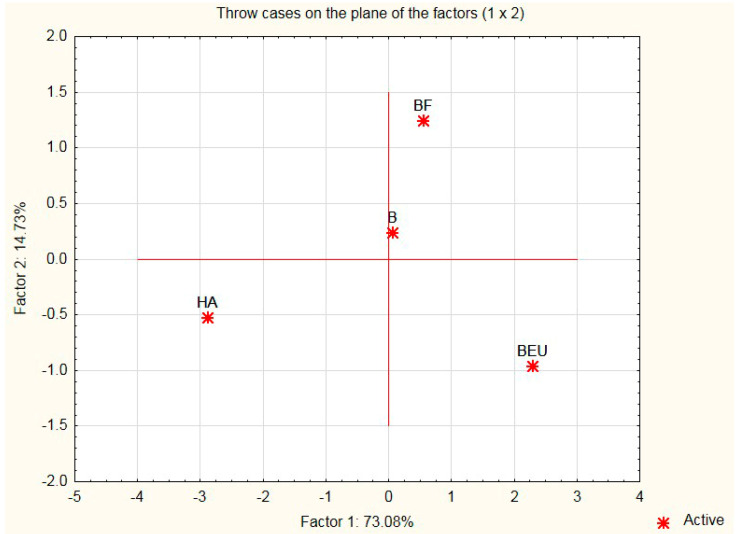
Projection of cases on the plane of factors.

**Figure 5:**
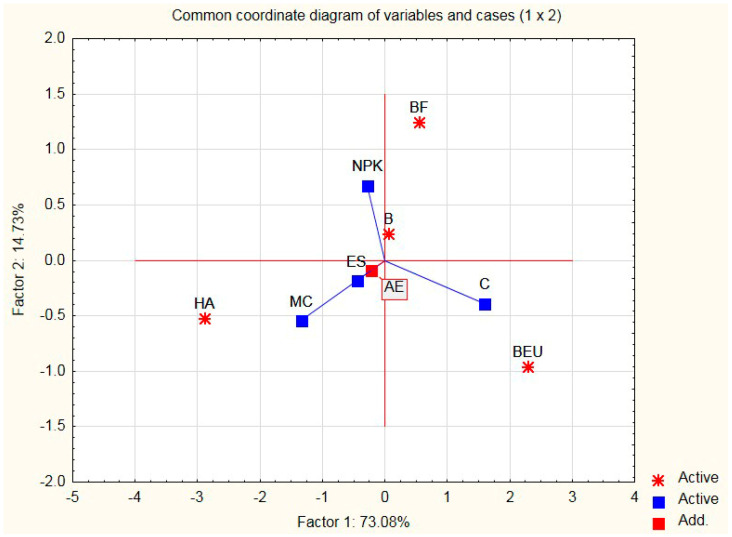
Projection of cases on the plane of factors (integrated).

Graphical interpretation makes it possible to determine the correlation between the simultaneously compared additives and their criteria. In the concept of multivariate analysis, it is presented by biplots (Figure 3, Figure 4 and Figure 5), which enable the graphical presentation of the row and column elements of a data table on the same graph [41,42]. For our graphical interpretation, a map of the assessment of the criteria (features) of additives was built on the basis of the principal components analysis method in the module “Principal Component Analysis and Classification” of the STATISTICA 10.0 package [43]. Data Table 1 and Table 2 present the medians of the evaluation of the additions (modifiers) of the variables (traits) to the silicate mass (sand–lime) and the cases (modifiers).

The above tables present 4 modifiers and 5 variables (criteria) representing measurable data and medians. The active variables for the analysis are those influencing the microstructural properties of sand–lime products (MC), economy (C), ecology (ES), and photosynthesis (NPK). It is true that the NPK variable is more appropriate for plants, but biohumous plants contain CaO, which affects the microstructure of sand–lime products; hence, it has little influence on the microstructure of the silicates. It will be presented on the biplot, although it will not take part in building the main axes. The biplot is built on the basis of a covariance matrix in order to obtain information about the differentiation of individual variables. The next stage of the work included determining the optimal dosage of the organic modifier in a liquid form (Humus Activ (HA), Biohumus Extra Universal (BEU)), and then the optimal composition of the sand–lime mass and the percentage composition of the modifier amount in relation to the mass in question. After selecting the variables and running STATISTICA 10.0 [43], the principal axes and the point positions representing the rows and columns of the data table were calculated (according to your software version). Graphical interpretation allows for distinguishing two main components, which are the main axes of the system, which explain almost 88% of the total variance of the 4 active variables taken for the analysis. In order to obtain a representation of variables and cases, a 2W plot of factor coordinates of variables is run, as well as a 2W plot of factor coordinates of cases.

The obtained map shows the location of the active (navy blue squares) and passive (red square) variables (vectors) and points representing modifiers for silicate products (stars) on a common coordinate system. Continuous lines show the vectors of variables: blue: active variables, and red: passive variable (Figure 3, Figure 4 and Figure 5).

### 2.2. Determination of the Composition of a Lime-Sand Mixture Modified with a Selected Organic Mo-Difier in a Liquid Form

The methodology of planning the experiment is presented in Table 3. A complete plan of 3 ** (2-0) was used, with the number of input quantities (independent variables: 2, the number of systems (measurements): 9, and the number of block 1.

**Figure 6:**
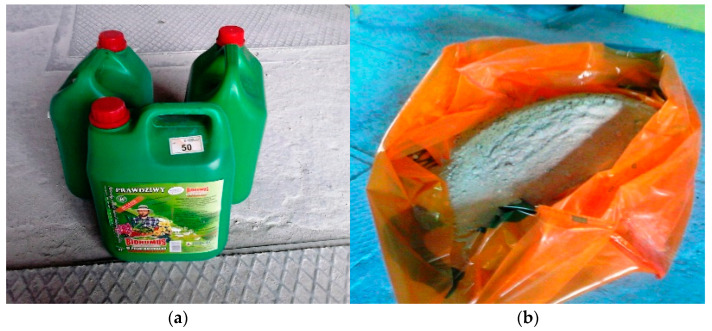
Substrates for the production of lime–sand brick modified with biohumus (biohumus in liquid form and quartz sand): (**a**) mix modifier used in the lime–sand mixture; and (**b**) modified sand–lime mixture.

In order to determine the effect of the effectiveness of the additive (mix modifier) application on the functional properties of products obtained from masses of various compositions, samples of autoclaved sand–lime products were each time obtained according to the same technology. This technology consists of the fact that after weighing the required amounts of the basic components of the mass, i.e., lime and sand (Figure 6b), they are mixed and then the lime slaking process takes place. After adding the modifier mix modifier (without water, Figure 6a) to this mixture and re-mixing it, the products are shaped and autoclaved.

Autoclaving temperature: approx. 203 °C.

Autoclaving time: 8 h.

The influence of the applied mix modifier on the quality of the product made of a properly prepared mass was assessed on the basis of a comparative analysis of traditional sand–lime products and products modified with an additive.

This stage of work included the performance of laboratory tests with the use of modifiers, which, as a result of technical and economic analysis, obtained the best suggested results. A series of sand–lime samples was made on the basis of selected organic modifiers in a liquid form (according to the above scheme).

**Figure 7:**
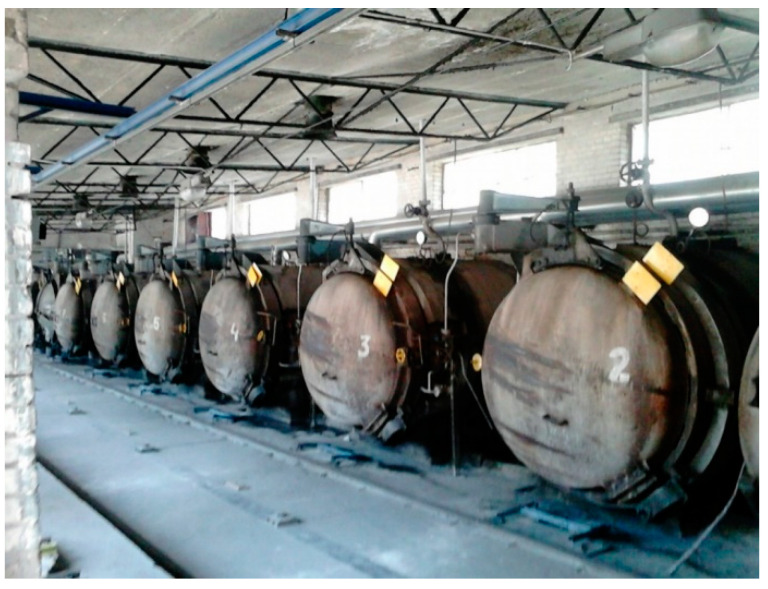
View of the production hall with autoclaves.

**Figure 8:**
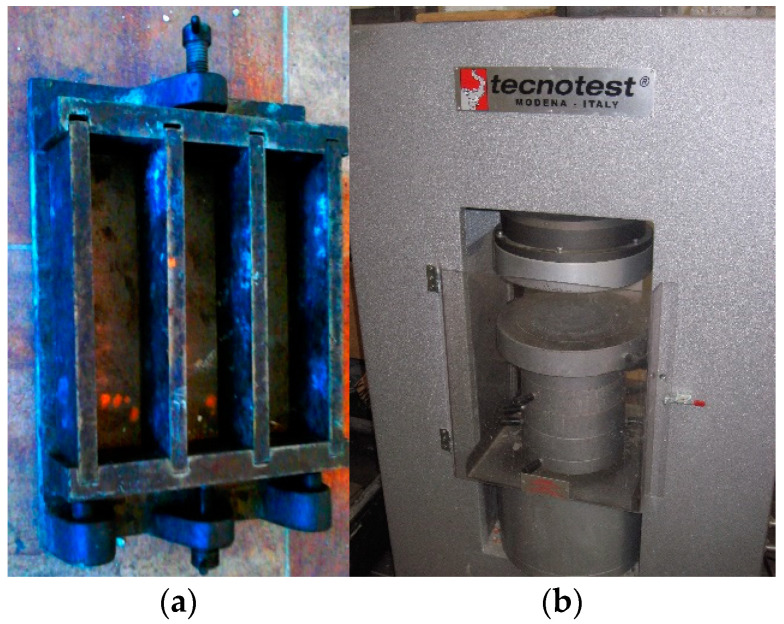
Form and method of sample preparation for laboratory testing of silicate products: (**a**) a three-chamber form for laboratory tests of sand–lime products; (**b**) hydraulic press for laboratory tests of compressive strength.

Laboratory samples, in the amount of 6 pieces for each number of tests, were made at the SILIKATY Production Plant in Ludynia (Poland) using the local lime–sand mixture and autoclaves (Figure 7) under the supervision of the chief technologist. Each sample was made in three-chamber molds with bar dimensions of 4 × 4 × 16 cm (Figure 8a). Each sample was thoroughly mixed and then compressed on a press in order to mechanically compact the modified sand–lime mass. First, a sample of the product was made without modification in order to properly verify and confront the data with the modified elements. After the laboratory samples were prepared, the molds were placed in autoclaves and subjected to a hydrothermal treatment process (Figure 7).

### 2.3. Laboratory Tests

Samples after autoclaving were subjected to laboratory tests aimed at determining to what extent the modification turned out to be favorable or unfavorable. All disassembled and prepared elements were examined, and the following tests were performed:

- Compressive strength using the Tecnotest KC 300 hydraulic press (Figure 8b);
- Bulk density;
- Absorption due to rising capillary;
- Pore size and arrangement;
- X-ray examinations.

In addition, the compressive strength test, which is related to the durability of the modified element, was confirmed by a test using a Schmidt hammer.

The developed composition of the lime–sand mixture modified with an organic modifier in a liquid form improves the process of mixing the mass, and the modifier can be easily combined with water while mixing the lime–silicate mass. First, a liquid modifier is added to the sand–lime mass in appropriate proportions (3, 7, and 11% of the additive or additives) in relation to the mass of the product, and then, when necessary (too dry mixture), water is added. Such a dosing process of the modifying substance is optimal for the company, because there is no need to modify the technological process of producing sand–lime products in line with the efficient use of energy and the proper process of sand–lime brick production.

**Figure 9:**
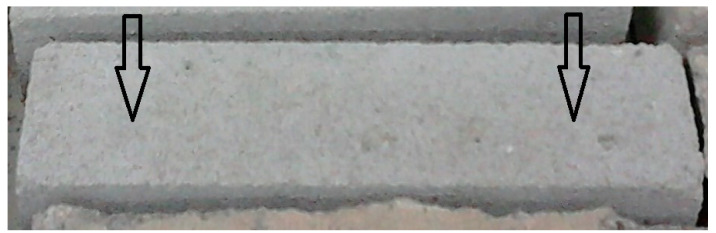
Compressive strength test of a laboratory beam with dimensions of 4 × 4 × 16 cm.

During the compressive strength test (Figure 9) each bar was tested from two sides and the force of pressure on the sample during its destruction was measured.

Another test performed was water absorption. Each sample to be analyzed was immersed in the so-called water. The grain surface is where the speed and method of water absorption were checked. The weight of the sample exposed to the water was then monitored until the H_2_O uptake was stopped. The sample was weighed and the % water absorption of the modified sand–lime element in relation to the traditional product was determined. As part of the analysis porosity of modified bricks was also performed (by SEM), the size and arrangement of pores in a modified sand–lime product was determined. The strength value depends on the proper compaction of the silicate mass, well-selected substrates, and appropriately formed structures in the microstructure of the autoclaved product. Hydrated calcium silicates, such as C-S-H, tobermorite, and yennite (with a high proportion of binder) are formed at the interface between the binder and sand, and then in the pores. The C-S-H phase is deficient in silicates, which is even more noticeable in the modified sample, because it is a metastable phase and crystallizes at a temperature higher than 30 °C. The dominant phase is tobermorite.

X-ray examinations were used to determine the actual structures and chemical compounds present in the tested materials. In the case of testing building materials, it is recommended to double-test one sample at different angles of incidence of the electron beam, or if this method is not possible, it is recommended to test the material twice by taking two separate samples from a similar series. The tests were performed on the basis of the applicable and mentioned norms and standards. The X-ray examination was performed due to the fact that organic substances were added to the lime–sand mixture, as well as to check the compounds that occur in the structure of the material during chemical reactions (the material must be ecological and safe to use).

## 3. Results

### 3.1. Compressive Strength and Bulk Density Testing

**Figure 10:**
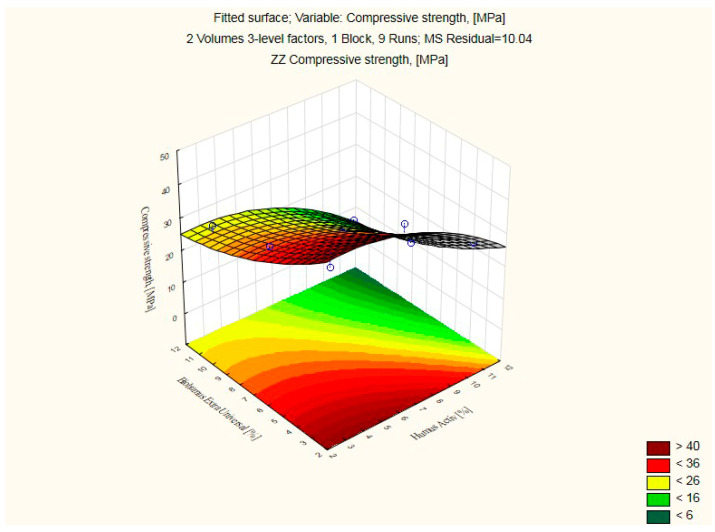
Response fitting surface plot for compressive strength of modified silicate products with Humus Activ and Biohumus Extra Universal.

**Figure 11:**
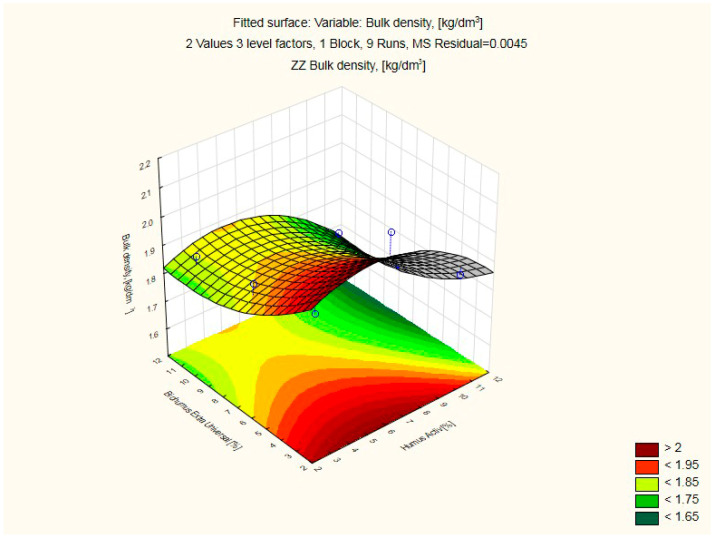
Response fitting surface plot bulk density of the modified silicate product with Humus Activ and Biohumus Extra Universal.

The results of the compressive strength of the silicate products modified with Humus Activ and Biohumus Extra Universal are shown in Table 4 and Table 5 and Figure 10, while the bulk density is presented in Table 4 and Table 6 and Figure 11. The samples with numbers 1, 2, 4 turned out to be the best in terms of increasing the compressive strength. Modification of the element with 10% of organic compounds positively influenced both the compressive strength of the product (42.04 MPa) and the bulk density (2.11 kg/dm^3^). For the control sample, the compressive strength was (20.51 Mpa), and the volume density (1.73 kg/dm^3^). The experiment is presented in the Table 4. Water absorption reached 9% and maintained this level after increasing the amount of modifier. The modifier in the liquid form did not change the porous structure of the material, but only reduced water absorption by about 56%. This is because the modifier has a liquid form (humus/biohumus), and silicate bricks, after filling the pores with water (or in this case with the modifier), reach the state of maximum water absorption and do not absorb liquids. Traditional silicate products are characterized by water absorption up to 16% in relation to the weight of the product.

As the analysis performed showed, positive changes occur in the modified products for compressive strength and bulk density.

### 3.2. Water Absorption Test

**Figure 12:**
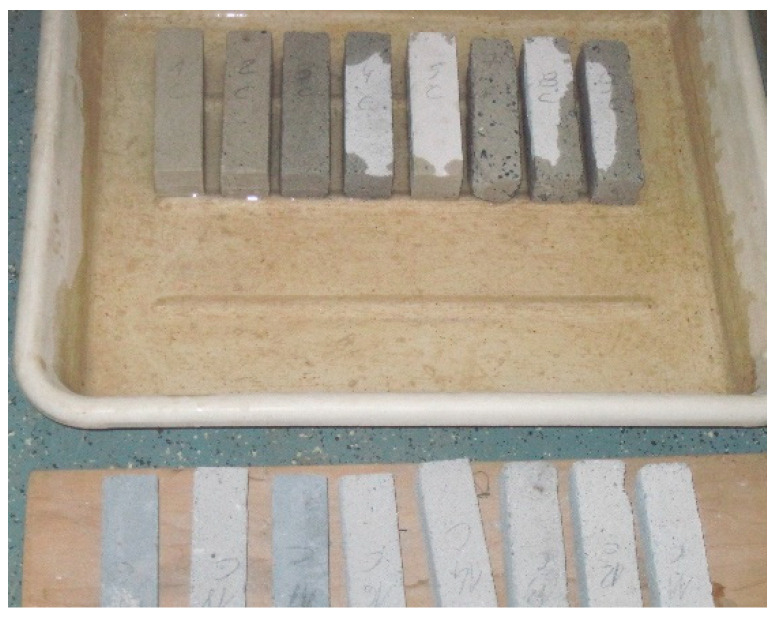
Study of the absorption caused by capillary rising of modified sand–lime products.

The modified silicate products were characterized by a much lower water absorption in the water absorption process. During the capillary rise test (water absorption), the samples absorbed water in an amount from 1/3 to ½ less than in a standard sand–lime product (Figure 12). The concept of capillary absorption is a phenomenon related to the transport of water coming from the ground up the wall. The visible effects of capillary rising are moisture in the lower parts of the wall, often in the form of horizontal dampness. When calculating the water absorption coefficient of modified sand–lime products caused by capillary rising, we used the following formula:

c_wi,s_ = [(m_so,s_ − m_dry,s_)/ A_s_t] × 10^3^ [kg/(m^2^·min)] (1)

where:

c_wi,s_—initial water absorption of masonry elements,
m_so,s_—mass of the sample in grams after soaking with water at time “t”,
m_dry,s_—mass of the sample after drying,
A_s_—total face area of the sample immersed in water [mm^2^],
t—water saturation time [s].

All samples with the modifier admixed absorbed water evenly and gradually (the samples were placed in a vessel with water with the facing surface). Modified silicate elements absorbed water for almost two times longer than their traditional counterparts.

As the analysis presented in Section 3.1 and Section 3.2. shows, changes occur in compressive strength and bulk density, while absorption does not undergo major modifications (and remains almost constant).

**Figure 13:**
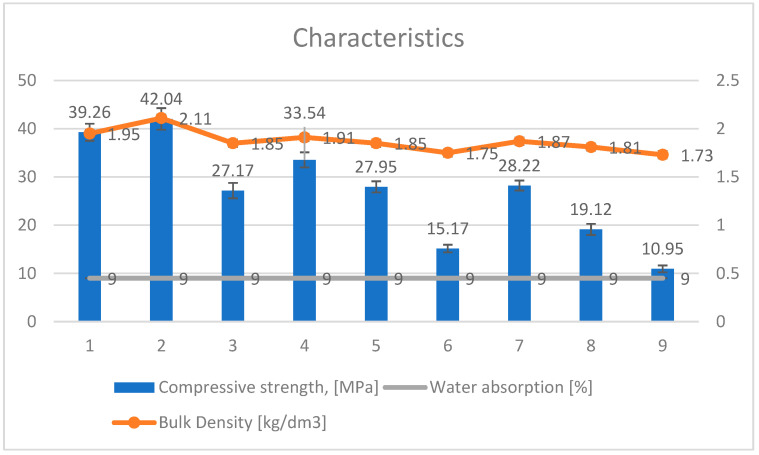
Dependence of compressive strength (red line), bulk density (blue line) and water absorption (green line) of silicate autoclaved materials modified with Humus Activ and Biohumus Extra Universal.

The compatibility of the relationship of the modified autoclaved material with Humus Activ and Biohumus Extra Universal is shown in Figure 13.

**Figure 14:**
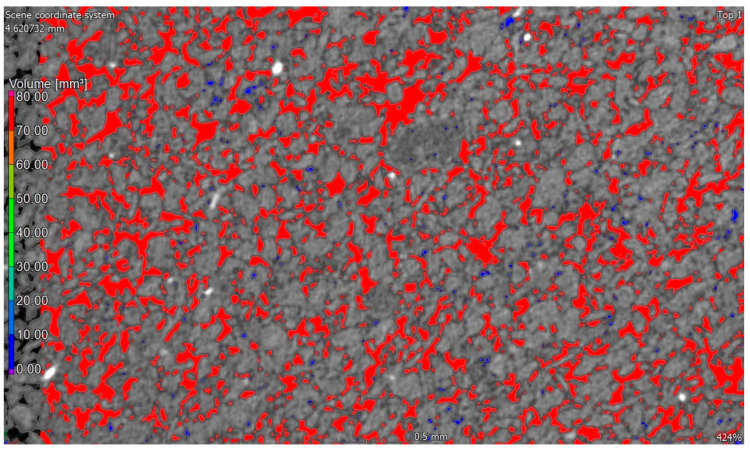
Image of the porosity of the silicone material made by CT. The diagram shows the layout and structure of the pores.

As the analysis showed, changes occurred in the compressive strength and bulk density, while water absorption was reduced to 9% and kept at a constant level (Figure 14). As mentioned, the modifier did not change the porous structure due to its liquid form, but due to its different density than water, it changed the physical and chemical parameters.

Micro-CT analysis was performed on a Nikon XT H 225 ST CT scanner (Minato-ku, Japan). The industrial computed tomograph is designed for non-destructive testing, as well as non-surface and visualization analyses, which enables an insight into the interior of the tested material.

### 3.3. Examination of the Microstructure of a Silicate Product

**Figure 15:**
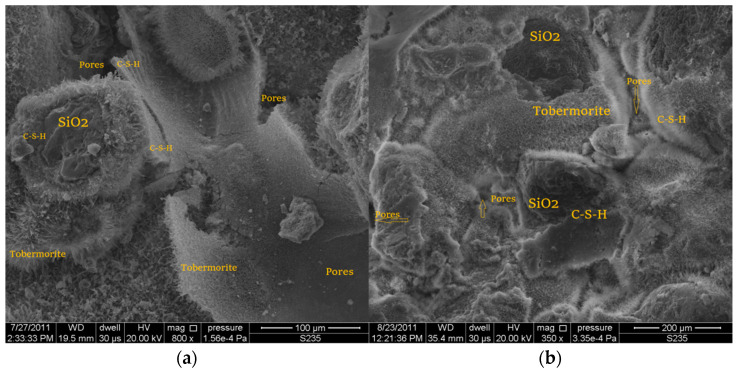
Scanning electron microscope (SEM) photos of the microstructure of silicate bricks: (**a**) the structure of traditional sand–lime products, mag.: 100 μm; and (**b**) structure of lime-sand products modified with 10% admixture in the form of a mix modifier, mag.: 200 μm.

The photo Figure 15a,b and Figure 14 show the internal structure of a sand–lime product with visible pores (dark, free spaces between the shaped forms). In traditional sand–lime products, the so-called C-S-H phase appears as an amorphous mass (resembling the structure of cotton wool or fibers).

Interference with the lime–sand mixture with an admixture in the form of a modifier mix resulted in an increase in compressive strength, which is related to the compaction of the pores in the product, which is also visible in the photo on the right. The photo (Figure 15b) shows a much smaller number of pores than in the traditional sample.

**Figure 16:**
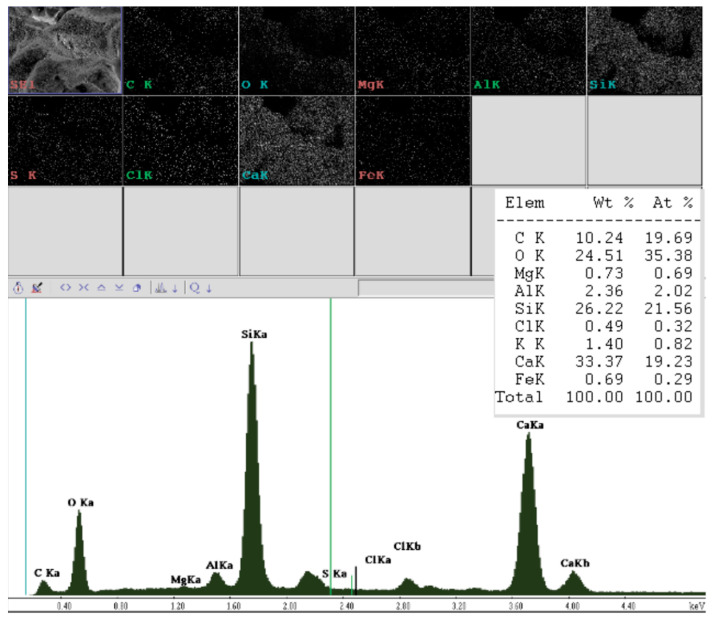
EDS spectrum, i.e., the elemental composition of the tested product modified with a predetermined admixture (the main components are: Si, O, and Ca). Al, Mg, and S are elements found in sand.

Figure 16 shows the elemental composition of silicate materials. As you can see, these are natural and environmentally friendly materials, and modification with humus does not affect their chemical composition.

### 3.4. X-ray Examination

**Figure 17:**
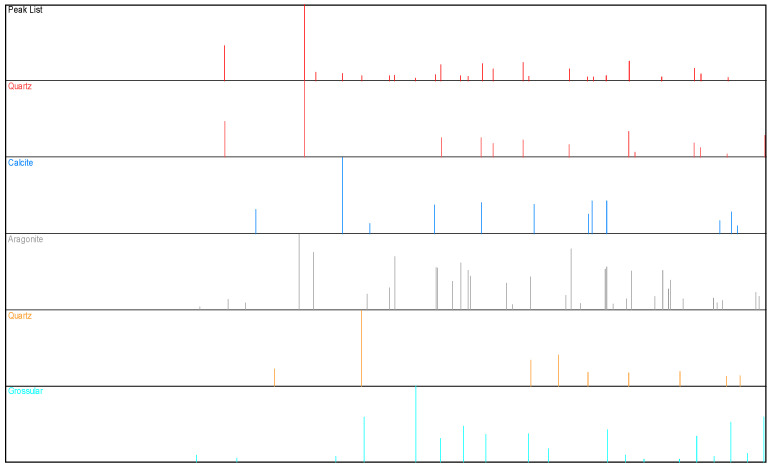
Structural X-ray (XRD) results of the autoclaved bricks.

The study showed the presence of minerals and compounds such as: Aragonite, quartz, calcite, grossular, aragonite, kwarc, or calcite (Figure 17), which are typical minerals found in the structure of a lime–sand product due to their composition (silicates of a different structure). Grossular also belongs to the group of silicates, but is extremely rare. Its presence may be related to an increase in the strength characteristics of the modified product. The highest peaks are observed from aragonite, then from calcite and quartz.

## 4. Usefulness of Modified Silicate Products

**Figure 18:**
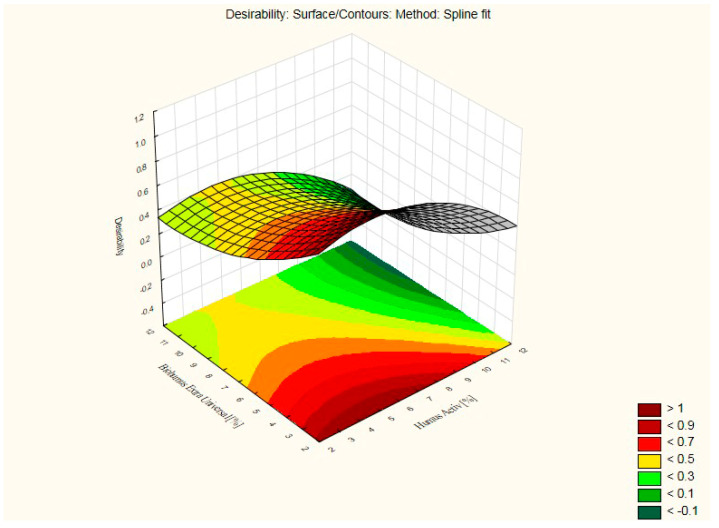
Graph of usability functions and its projection, with points of total usefulness (compressive strength and bulk density) of silicate products with the addition of Humus Activ and Biohumus Extra Universal.

Based on the theoretical literature considerations presented, the laboratory tests performed, and the analyses using mathematical experimental planning, the average and optimum material solutions of the modified silicate products were determined. The desired values of the input variables (Humus Activ and Biohumus Extra Universal) for the output variables (compressive strength and bulk density) were determined. Considering the practical applications for modified silicate products, the minimum value of the output variables should be higher than the value for traditional products, while the maximum value should be expected near the satisfactory value at a high level. Therefore, for compressive strength in this case, a range of [20–40 MPa] was adopted, and for bulk density, a range of results is included in Table 4, [1.73–2.11 kg/dm^3^]. Satisfactory values were set at three (3) levels, as shown in Table 7 and Table 8:

Figure 18 shows a graph of usability functions and its projection, with points of total usefulness (compressive strength and bulk density) of silicate products with the addition of Humus Activ and Biohumus Extra Universal.

**Figure 19:**
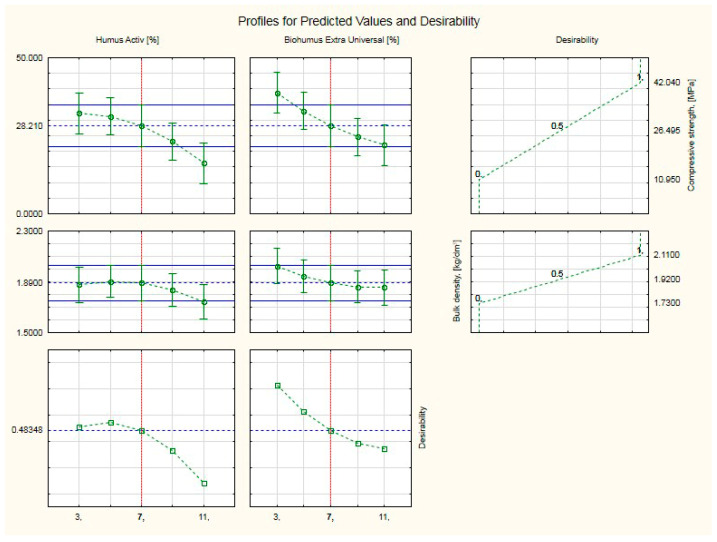
Profiles of predicted values and desirability. Profile of overall usability (compressive strength and bulk density) of silicate products with Humus Activ and Biohumus Extra Universal, for average values.

Figure 19 shows profiles of predicted values and desirability. Profiles of overall usability (compressive strength and bulk density) of silicate products with Humus Activ and Biohumus Extra Universal, for average values. The values of the input variables, i.e., for Humus Activ and Biohumus Extra Universal, were set at average values, the same for both variables—7%. A desirability of 0.48 was obtained, which classifies the result at a satisfactory average level, with compressive strength (28.21 MPa) and bulk density (1.89 kg/dm^3^), i.e., above the values for the control silicates (compressive strength was (20.51 MPa), and the bulk density (1.73 kg/dm^3^).

**Figure 20:**
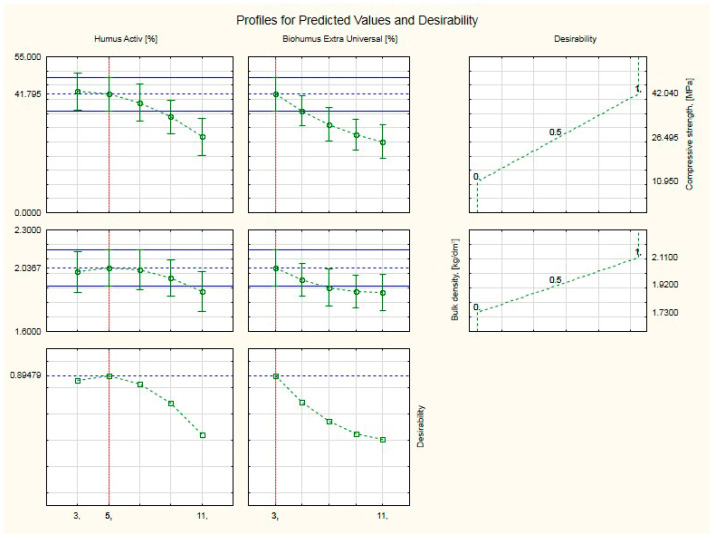
Profiles of predicted values and desirability. Profile of overall usability (compressive strength and bulk density) of silicate products with Humus Activ and Biohumus Extra Universal, for optimum values across the range.

Figure 20 shows profiles of predicted values and desirability. Profiles of overall usability (compressive strength and bulk density) of silicate products with Humus Activ and Biohumus Extra Universal, for optimum values across the range. The values of the input variables were set at their expected optimum values, i.e., 5% for Humus Activ and 3% for Biohumus Extra Universal. A desirability of 0.89 was obtained, which classifies the result as satisfactorily high, with compressive strength (41.80 MPa) and bulk density (2.04 kg/dm^3^), i.e., above the values for the control silicates (compressive strength was (20.51 MPa), and the bulk density (1.73 kg/dm^3^).

## 5. Conclusions

This the aim of the article was to investigate the possibility of using humus and vermicompost, i.e., organic matter resulting from a long process of biological decomposition, in the production of autoclaved bricks containing only ecological materials, i.e., sand, lime, and water. The essence of this solution consists in the use of an organic modifier (mix modifier) in an amount below 14% introduced into the sand–lime mass. Introducing an additive in the form of an organic modifier (mix modifier) to the traditional sand–lime mass at the production stage has a positive effect on the course of chemical processes taking place during the autoclaving process of the discussed products, which in turn significantly improves the compressive strength of the silicate product. The addition of a mix modifier reduces water absorption, as it limits the access of water to the interior of the modified product. The compressive strength increased to 42.04 MPa (compared to standard bricks, whose strength is 15–20 MPa), and the bulk density increased by about 55% to the value of 2.11 kg/dm^3^, which indicates the densification of the material’s microstructure. Below is the most important information to prove the purpose. This goal is achieved by adding Humus Activ and Biohumus Extra Universal to the previously prepared lime–sand mass in the amount of 6–14% of individual components.

The advantages of silicate brick are:

- Increased compressive strength of silicate brick. Modifying the element with 10% of organic compounds favorably influenced both the compressive strength of the product (42.04 MPa) and the bulk density (2.11 kg/dm^3^). For the control sample, the compressive strength was (20.51 MPa), and the volume density (1.73 kg/dm^3^);
- Improved performance properties (reduced water absorption, during the capillary rise test (water absorption). The samples absorbed water in an amount from 1/3 to ½ less compared to a standard sand–lime product);
- The microstructure of the silicate product indicates the presence of the tobermorite phase as the main phase and the C-S-H phase as the deciphyte phase. Under the influence of autoclaving, high temperature, and pressure in the autoclave, the C-S-H phase usually crystallizes in the direction of tobermorite, which is also related to the amount of binder in the material.

The composition of humus and biohumus introduced into the silicate mass may cause some problems due to its composition (Figure 1) because the percentages of individual oxides may slightly differ depending on the product batch despite the quality analysis. Looking at the elemental composition, however, it can be concluded that the crystallization of the C-S-H phase under the influence of hydrothermal conditions will continue towards the tobermorite phase—this is evidenced by the low content of binder (CaO), and the presence of Al_2_O_3_ in sand and biohumus leads to the synthesis of the tobermorite phase. On the other hand, the presence of Fe_2_O_3_ in the biohumus positively affects the strength properties of the modified silicate brick. Similarly to the results of Abdoliyazdi and all or Largeau [44,45], the mechanical properties of samples containing 1 and 3 percent of Fe_2_O_3_ nanoparticles are more desirable than those of ordinary cement mortar. SEM studies of the microstructure of the cement mortar containing nanoparticles and the ordinary cement mortar showed that Fe_2_O_3_ nanoparticles completely fill the pores and reduce large Ca(OH)_2_ crystals, and the hydrate products are denser and compact (increasing the proportion of Fe_2_O_3_ to 5% reduces the mechanical properties according to Nooshin Abdoliyazdi and all. The above thesis was also confirmed in the modification of the silicate mass with biohumus and humus (there was an increase in strength values, an improvement in density due to the compaction of the structure and a reduction in water absorption). Probably, the increase in the content of CaO in the raw material mass due to the participation of modifiers potentially caused crystallization in the direction of jennite, and thus changes the technical and operational properties of silicate products. To confirm this thesis, the next study will be XRF analysis and thermodynamic analysis using geochemical modeling.

- Can be used as foundation blocks;
- Classifying the silicate product as a sustainable material (material in the form of aggregate can be recovered after the expected period of use);
- There were opportunities to improve the technological process of producing silicate products in terms of effective use of energy, thanks to the use of mix modifiers in liquid form.

## Figures and Tables

**Table 1:** Characteristics of organic silicate mass fillers for technical and economic analysis in measurable and non-measurable values.

Type of Modifier (Cases)	Criteria (Features) Variables				
	Microsilica Content (MC) [kg/dm^3^]	Cost (C) [euro/L]	Content NPKCaO (NPK) [mg/L]	Ecological (ES) Sustainability	Aesthetics (AE)
Humus Activ (prod. EKODARPOL)	Yes	3.3	800, 300, 300, 300	sustainable development	dark gray
Biohumus Extra Universal (prod. EKODARPOL)	No/Yes	2.0	700, 300, 300, 1100	environmentally friendly	dark gray to black
Biohumus Forte (prod. AGRECOL)	No	2.9	800, 500, 1200	environmentally friendly	dark gray to black
Biohumus (prod. FERTY-GREEN)	No	2.4	700, 300, 300	environmentally friendly	dark gray to black

**Table 2:** Characteristics of organic silicate mass fillers for technical and economic analysis in median values.

	Criteria (Features) Variables				
Type of Modifier (Cases)	Micro-Silica Content [kg/dm^3^]	Costs [euro/L]	NPK Participation	Sustainable Ecology	Aesthetics
Humus Activ (HA) (prod. EKODARPOL)	5	1	4	5	2
Biohumus Extra Univ (BEU) (prod. EKODARPOL)	2	5	3	4	1
Biohumus Forte (BF) (prod. AGRECOL)	2	3	5	4	1
Biohumus (B) (prod. FERTY-GREEN)	2	2	3	4	1

**Table 3:** Experiment planning matrix and levels of independent factors.

№	Independent Factors			
	On a Contractual Scale		On a Natural Scale	
	X_1_	X_2_	X_1_ (HA) (Humus Activ) [%]	X_2_ (BEU) (Biohumus Extra Universal) [%]
1	−1	−1	3	3
2	0	−1	7	3
3	1	−1	11	3
4	−1	0	3	7
5	0	0	7	7
6	1	0	11	7
7	−1	1	3	11
8	0	1	7	11
9	1	1	11	11

**Table 4:** Test results for the modification of sand–lime products with organic compounds.

№	Independent Factors		Output Variables		
	X_1_ (HA) (Humus Activ) [%]	X_2_ (BEU) (Biohumus Extra Universal) [%]	Compressive Strength, [Mpa]	Bulk Density [kg/dm^3^]	Water Absorption [%]
1	3	3	39.26	1.95	For samples, 9%—the modifier is in a liquid form, so the level of water absorption was reduced to 9% as a result of the modification. 16
2	7	3	42.04	2.11	
3	11	3	27.17	1.85	
4	3	7	33.54	1.91	
5	7	7	27.95	1.85	
6	11	7	15.17	1.75	
7	3	11	28.22	1.87	
8	7	11	19.12	1.81	
9	11	11	10.95	1.73	
10	-	-	20.51	1.73	

**Table 5:** Values of the studied factors at optimal points examined variable (compressive strength) and the respective objective function.

Variable (Criterion)	Optimal Point Coordinates		The Value of the Objective Function, [MPa]
	Humus Activ, [%]	Biohumus Extra Universal, [%]	
Compressive strength [σ]	3.01	14.47	24.37

**Table 6:** Values of the studied factors at optimal points examined variable (bulk density) and the respective objective function.

Variable (Criterion)	Optimal Point Coordinates		The Value of the Objective Function, [MPa]
	Humus Activ, [%]	Biohumus Extra Universal, [%]	
Bulk density [ρ]	5.33	10.33	1.87

**Table 7:** Ranges of satisfactory values for the criterion of optimization (compressive strength) of modified silicate product with Humus Activ and Biohumus Extra Universal.

Criterion	Range of Satisfying Values		
	Low U = 0.00	Medium U = 0.5	High U = 1.0
Compressive strength [MPa]	20	30	40

**Table 8:** Ranges of satisfactory values for the criterion of optimization (bulk density) of modified silicate product with Humus Activ and Biohumus Extra Universal.

Criterion	Range of Satisfying Values		
	Low U = 0.00	Medium U = 0.5	High U = 1.0
Bulk density [kg/dm^3^]	1.73	1.92	2.11

## Data Availability

Not applicable.

## Abbreviations

X1 (HA)	Humus Activ [%]
X2 (BEU)	Biohumus Extra Universal [%]
σ	Compressive strength [MPa]
ρ	Bulk density [kg/dm^3^]
c_wi,s_	Water absorption [%]
SEM	Scanning electron microscope
XRD	X-ray crystallography

## References

[1] Ullah A., Ali M., Shahzad K., Ahmad F., Iqbal S., Rahman M.H.U., Ahmad S., Iqbal M.M., Danish S., Fahad S. (2020). Impact of Seed Dressing and Soil Application of Potassium Humate on Cotton Plants Productivity and Fiber Quality. Plants.

[2] Zapotoczna-Sytek G. (2012). Naturalna promieniotwórczość wyrobów budowlanych, w tym autoklawizowanego betonu komórkowego (ABK). Przegląd Bud..

[3] Fiormarkets (2022). ‘Global Silica Brick Market’ Research Report. Global Silica Brick Market, Forecast to 2030. Fior Mark. Res..

[4] Kołacz B. (2020). Znaczenie Materii Organicznej W Glebie Oraz Działania Agrotechniczne Wspomagające Jej Utrzymanie.

[5] Kononova M.M. (1958). The Soil’s Humic Substances–Results and Problems in Humus Research. https://www.ecofarmingdaily.com/build-soil/humus/building-humus-for-all-crops/.

[6] Aytekin A., Aynur A.O. (2008). Hormone and Microorganism Treatments in the Cultivation of Saffron (*Crocus Sativus* L.). Plants. Molecules.

[7] Hussain N., Abbasi S.A. (2018). Efficacy of the Vermicomposts of Different Organic Wastes as “Clean” Fertilizers: State-of-the-Art. Sustainability.

[8] Allaga H., Bóka B., Nagy V.D., Szűcs A., Stankovics I., Takó M., Manczinger L., Vágvölgyi C., Kredics L., Körmöczi P. (2020). Composite Bioinoculant Based on the Combined Application of Beneficial Bacteria and Fungi. Agronomy.

[9] Jakubus M.A. (2020). Comparative Study of Composts Prepared from Various Organic Wastes Based on Biological and Chemical Parameters. Agronomy.

[10] Vitti A., Elshafie H.S., Logozzo G., Marzario S., Scopa A., Camele I., Nuzzaci M. (2021). Physico-Chemical Characterization and Biological Activities of a Digestate and a More Stabilized Digestate-Derived Compost from Agro-Waste. Plants.

[11] Kowalska A., Grobelak A., Almås A.R., Singh B.R. (2020). Effect of Biowastes on Soil Remediation, Plant Productivity and Soil Organic Carbon Sequestration: A Review. Energies.

[12] Szufa S., Piersa P., Adrian Ł., Sielski J., Grzesik M., Romanowska-Duda Z., Piotrowski K., Lewandowska W. (2020). Acquisition of Torrefied Biomass from Jerusalem Artichoke Grown in a Closed Circular System Using Biogas. Plant Waste. Molecules.

[13] Sojka M., Saeid A. (2022). Chapter 10—Bio-based products for agriculture. Smart Agrochemicals for Sustainable Agriculture.

[14] Pidlisnyuk V., Herts A., Khomenchuk V., Kononchuk O., Ust’ak S. (2021). Dynamic of Morphogical and Physiological Parameters and Variation of Soil Characteristics during Miscanthus giganteus Cultivation in the Diesel-Contaminated. Land. Agron..

[15] Lian H., Wang Z., Li Y., Xu H., Zhang H., Gong X., Qi H., Jiang Y. (2022). Straw Strip Return Increases Soil Organic Carbon Sequestration by Optimizing Organic and Humus Carbon in Aggregates of Mollisols in Northeast China. Agronomy.

[16] Tripolskaja L., Kazlauskaite-Jadzevice A., Baksiene E., Razukas A. (2022). Changes in Organic Carbon in Mineral Topsoil of a Formerly Cultivated Arenosol under Different Land Uses in Lithuania. Agriculture.

[17] Blakemore R.J. (2018). Critical Decline of Earthworms from Organic Origins under Intensive, Humic SOM-Depleting Agriculture. Soil Syst..

[18] Korkina I.N., Vorobeichik E.L. (2021). Non-typical degraded and regraded humus forms in metal-contaminated areas, or there and back again. Geoderma.

[19] Vogel S., Bönecke E., Kling C., Kramer E., Lück K., Nagel A., Philipp G., Rühlmann J., Schröter I., Gebbers R. (2020). Base Neutralizing Capacity of Agricultural Soils in a Quaternary Landscape of North-East Germany and Its Relationship to Best Management Practices in Lime Requirement Determination. Agronomy.

[20] Watabe A., Fukuda K., Saito T. (1987). Patent 62-090530—Soil Activator.

[21] Charier G.A., Le Guerinel H., Raimbault S., Jeanneau S. (2002). Organo-Mineral Soil Amendment Comprising Synergistic Mixture of Lime-Treated Household Rubbish and Plant Compost, Having e.g., Structuring, Clay-Humus Complex Formation and Aeration Promoting Effects.

[22] Nebehaj I., Venesz B., Odor G., Csapo F., Szuecs L. (1991). Process for Producing Biohumus Withincreased Microelement Content.

[23] Stepien A. (2021). Analysis of Porous Structure in Autoclaved Materials Modified by Glass Sand. Crystals.

[24] Kostrzewa-Demczuk P., Stepien A., Dachowski R., Krugiełka A. (2021). The use of basalt powder in autoclaved brick as a method of production waste management. J. Clean. Prod..

[25] Stepien A., Potrzeszcz-Sut B., Prentice D.P., Oey T.J., Balonis M. (2020). The Role of Glass Compounds in Autoclaved Bricks. Buildings.

[26] Borek K., Czapik P., Dachowski R. (2020). Recycled Glass as a Substitute for Quartz Sand in Silicate Products. Materials.

[27] Stepien A., Leśniak M., Sitarz M.A. (2019). Sustainable Autoclaved Material Made of Glass Sand. Buildings.

[28] Stepien A., Kostrzewa P., Dachowski R. (2019). Influence of barium and lithium compounds on silica autoclaved materials properties and on the microstructure. J. Clean. Prod..

[29] Stepien A., Kostrzewa P. (2019). The impact of basalt components on the structure of bricks formed as a result of hydrothermal treatment. Buildings.

[30] Pytel Z., Małolepszy J. (1990). Wykorzystanie błota pochromowego w produkcji wyrobów wapienno-piaskowych. Mater. Bud..

[31] Malhotra S.K., Tehri S.P. (1996). Development of bricks from granulated blast furnace slag. Constr. Build. Mater..

[32] Witthohn M., Klemm R. Proces zwiększania pojemności cieplnej cegieł wapienno-silikatowych oraz cegła z materiału wapienno-silikatowego. https://patents.google.com/patent/PL1752509T3/fr.

[33] Bavasso I., Vilardi G., Sebastiani D., Di Giulio A., Di Felice M., Di Biase A., Miliziano S., Di Palma L. (2020). Rapid Experimental Procedure to Assess Environmental Compatibility of Conditioning Mixtures Used in TBM-EPB Technology. Appl. Sci..

[34] (2011). 2011E: Metody badań elementów murowych. Część 1 Określenie wytrzymałości na ściskanie, Wymagania dotyczące elementów murowych. Część 2. Elementy murowe silikatowe.

[35] (2001). 2001 Methods of Test for Masonry Units-Part 13: Determination of the Density of the Net and Gross Density of Masonry in the Dry State (Except for Natural Stone).

[36] (2010). 2010 Eurocode 6-Design of Masonry Structures-Part 2: Design, Selection of Materials and Execution of Masonry.

[37] (2010). Specification for Masonry Units. Part 2: Calcium Silicate Masonry Units.

[38] Nermend K. (2020). Metody Analizy Wielokryterialnej i Wielowymiarowej We Wspomaganiu Decyzji. Wydawnictwo Naukowe PWN. https://ksiegarnia.pwn.pl/Metody-analizy-wielokryterialnej-i-wielowymiarowej-we-wspomaganiu-decyzji,734403196,p.html.

[39] Szafranko E. (2017). Applicability of multi-criteria analysis methods for the choice of material and technology solutions in building structures. Teh. Vjesn..

[40] Szafranko E. (2017). Application of multi-criterial analytical methods for ranking environmental criteria in an assessment of a development project. J. Ecol. Eng..

[41] Sagan A. (2004). Jeden obraz ukazuje więcej niż 10 liczb, czyli jak budować mapy zadowolenia klienta z wykorzystaniem programu Statistica. Statsoft Pol..

[42] Gabriel K.R., Barnett V. (1981). Biplot display of multivariate matrices for inspection of data and diagnois. Intrepreting Multivariate Data.

[43] (1989). Pakiet Oprogramowania do Zaawansowanej Analizy Danych.

[44] Abdoliyazdi N., Arefi M.R., Mollaahmadi E., Abdollahi B. (2011). To study the effect of adding Fe_2_O_3_ nanoparticle on the morphology properties and microstructure of cement mortar. Life Sci. J..

[45] Largeau M.A., Mutuku R., Thuo J. (2018). Effect of Iron Powder (Fe_2_O_3_) on Strength, Workability, and Porosity of the Binary Blended Concrete. Open J. Civ. Eng..

